# Supplementary material for: Changes in hospitalizations and emergency department respiratory viral diagnosis trends before and during the COVID-19 pandemic in Ontario, Canada
Source: PLoS One. 2023 Jun 16;18(6):e0287395. doi: 10.1371/journal.pone.0287395 (PMC10275476; doi:10.1371/journal.pone.0287395)

# S1 Fig: Age histogram by virus and hospital visit type

## A) Influenza virus


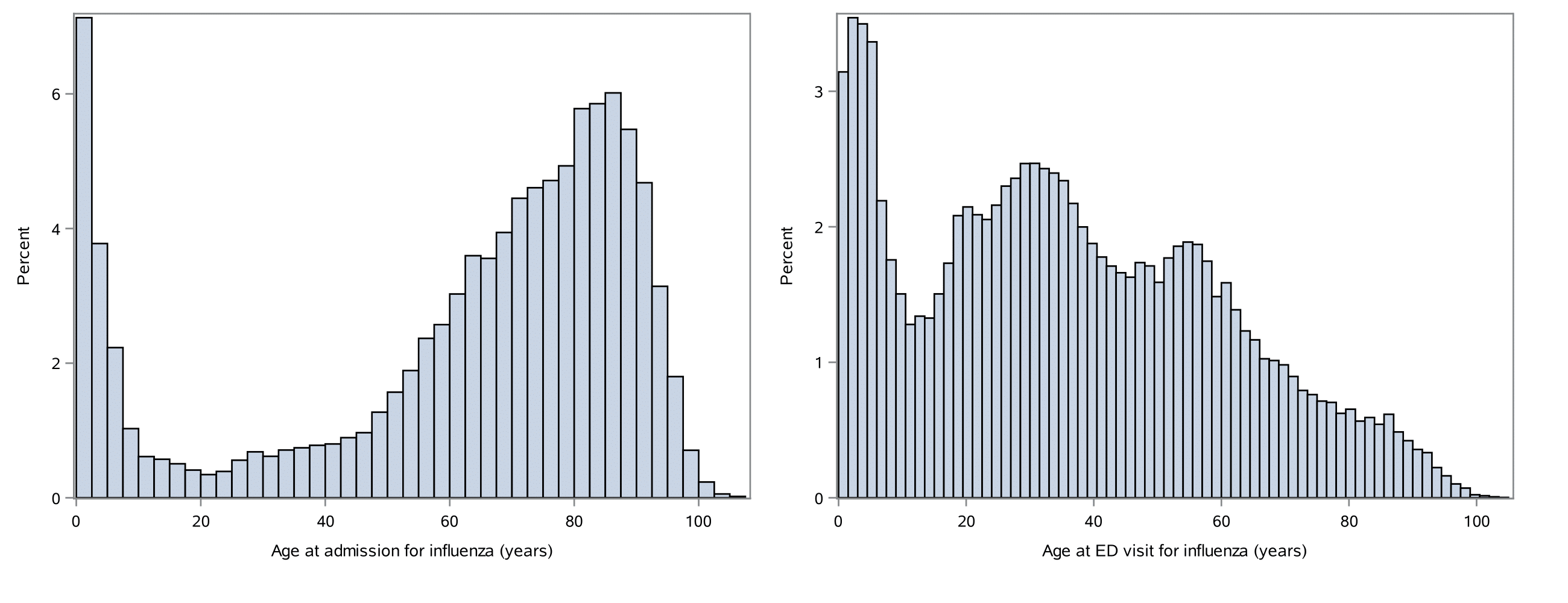


## B) Respiratory syncytial virus


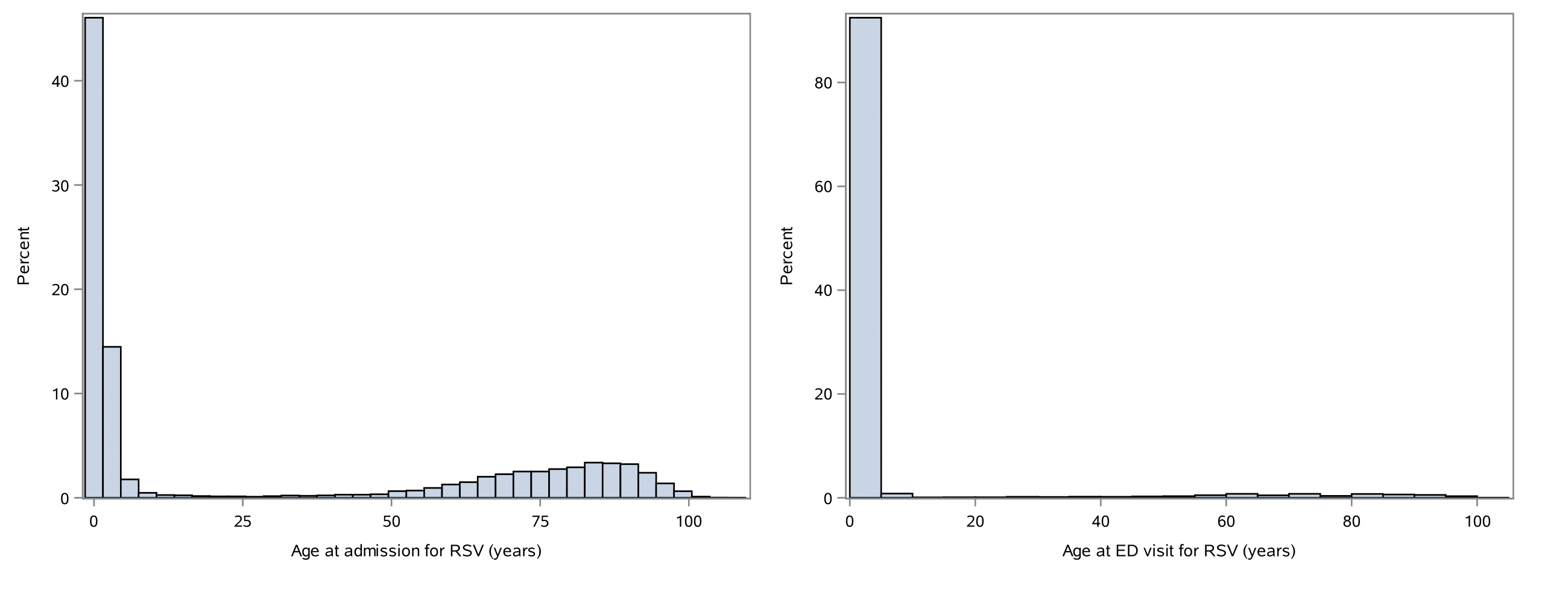


## C) Human metapneumovirus


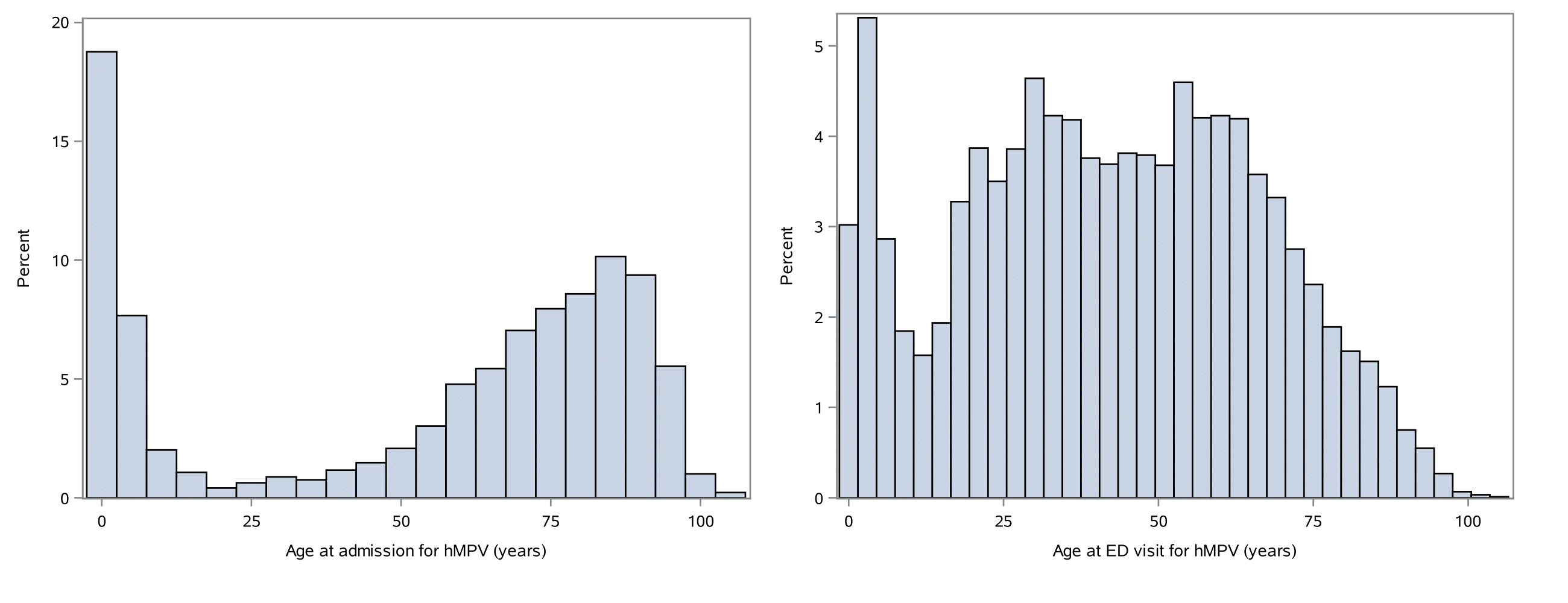


## D) Rhinovirus and enterovirus


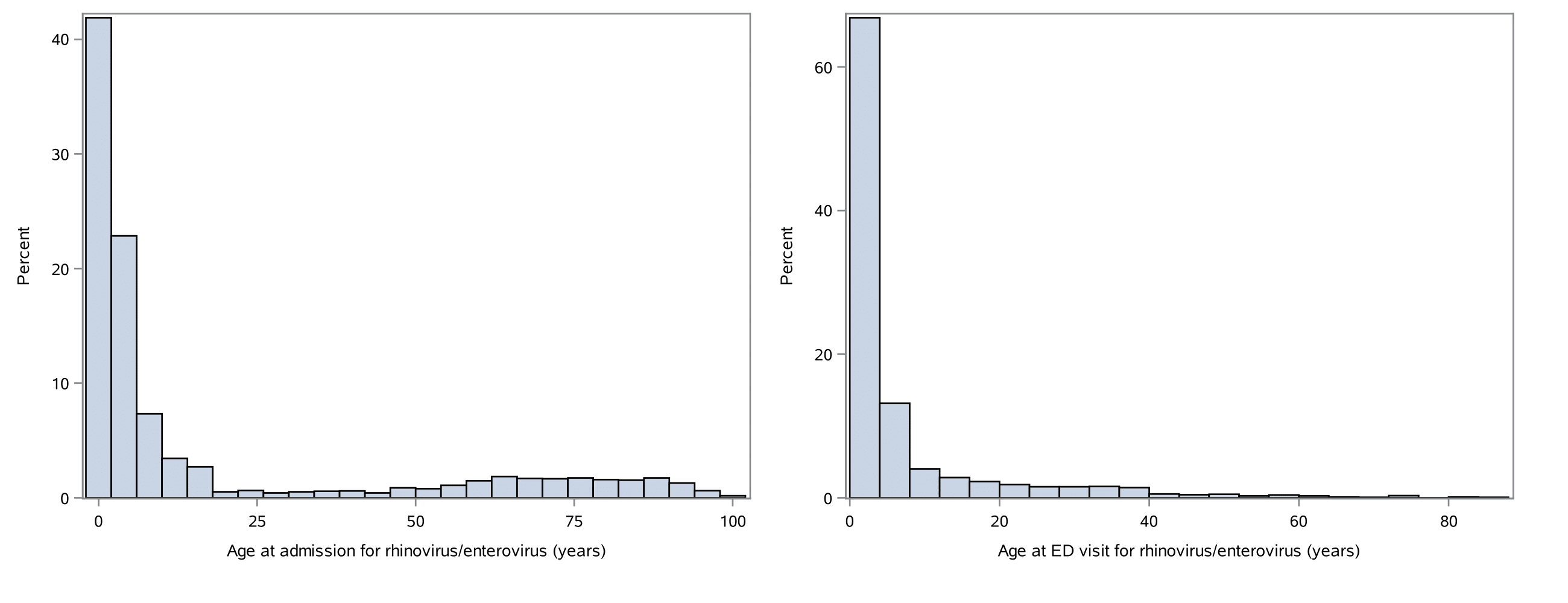


## E) Human parainfluenza virus


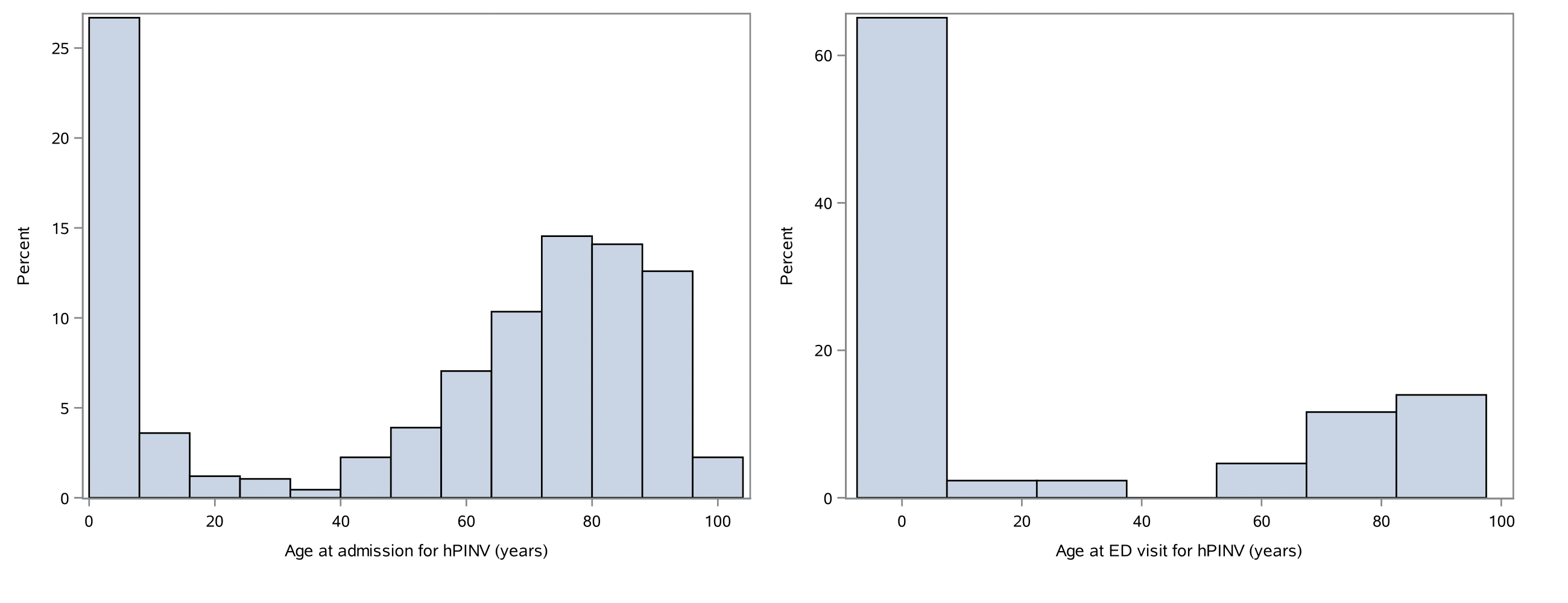


## F) Adenovirus


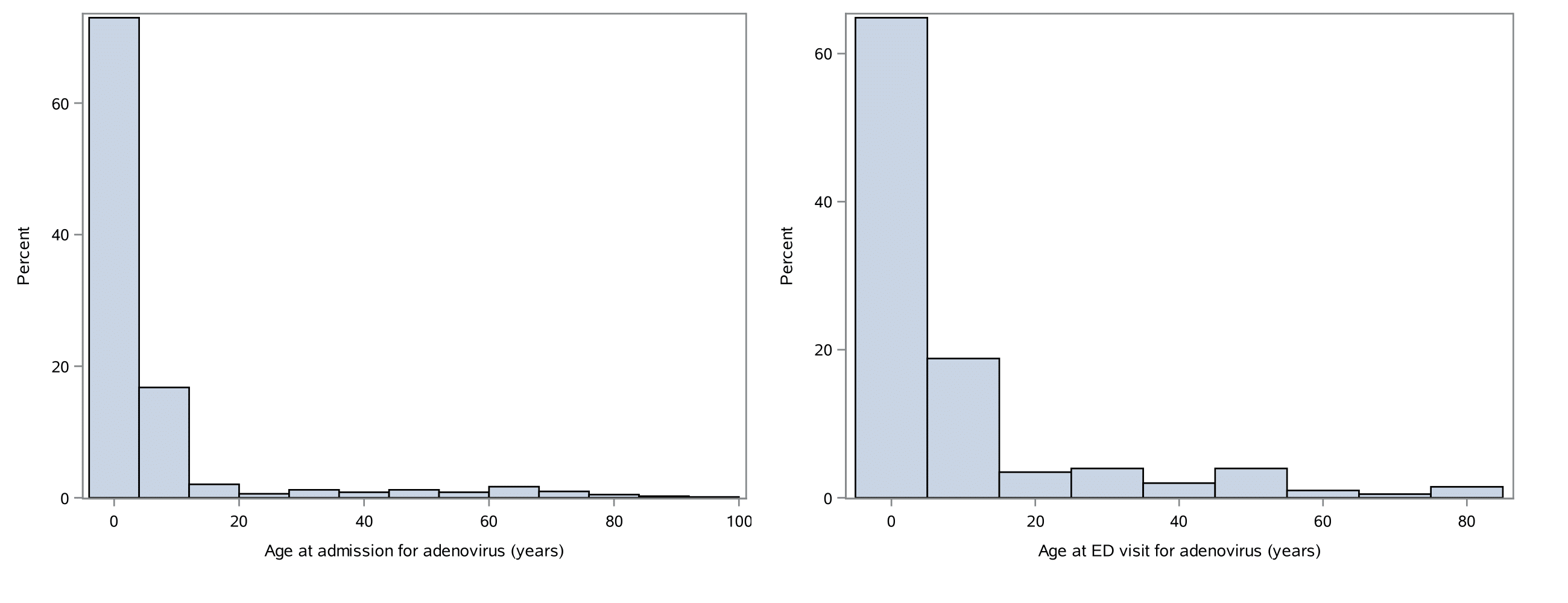


## G) Common cold coronavirus


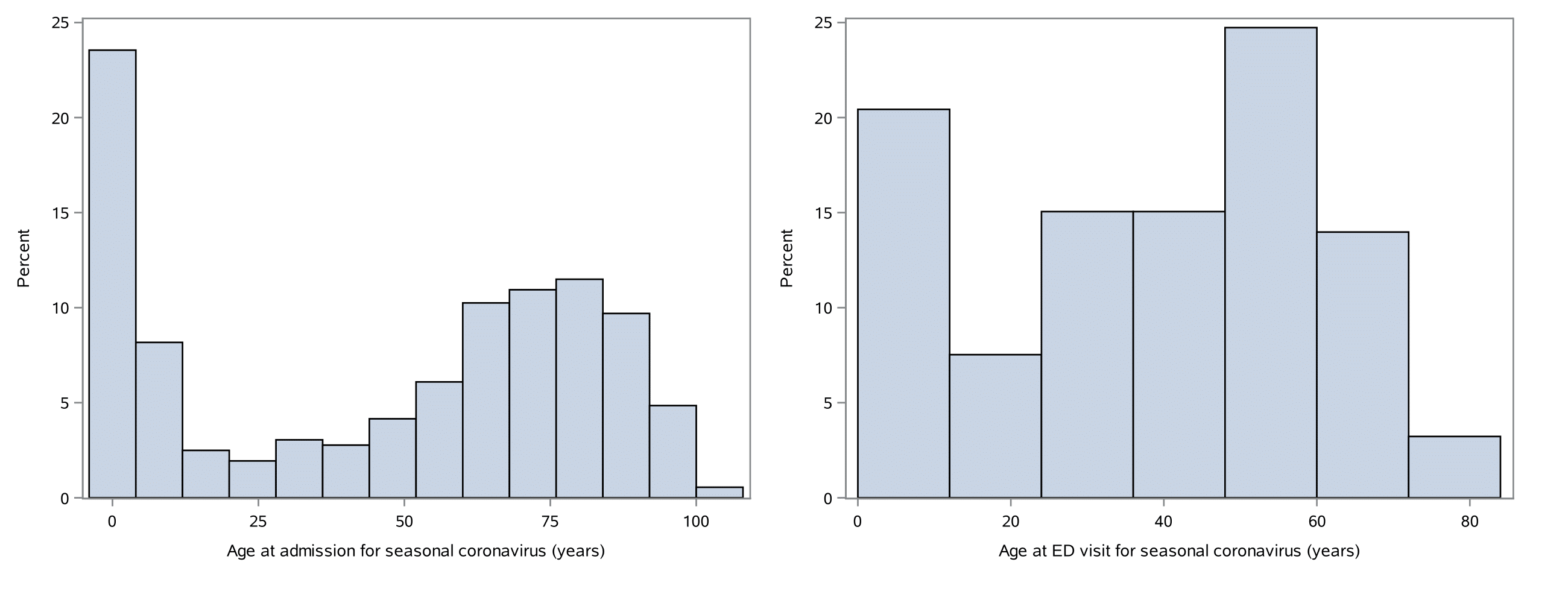

Supplement: S1 Fig — Histogram of age at admission or emergency department (ED) visit over the entire study period by virus diagnosis. (DOCX) [file pone.0287395.s001.docx]
