# Supplementary figures and images for: Changes in hospitalizations and emergency department respiratory viral diagnosis trends before and during the COVID-19 pandemic in Ontario, Canada
Source: PLoS One. 2023 Jun 16;18(6):e0287395. doi: 10.1371/journal.pone.0287395 (PMC10275476; doi:10.1371/journal.pone.0287395)

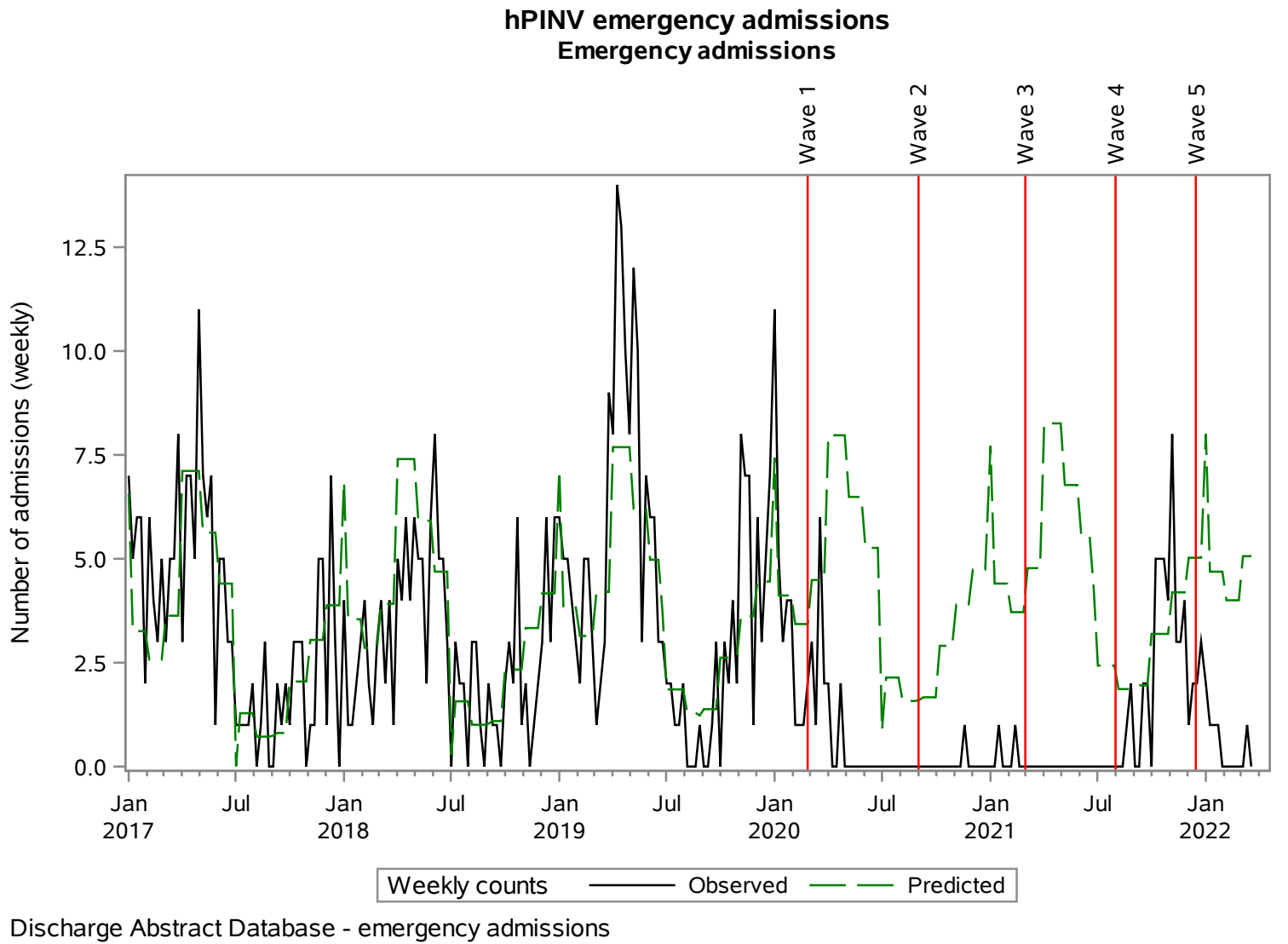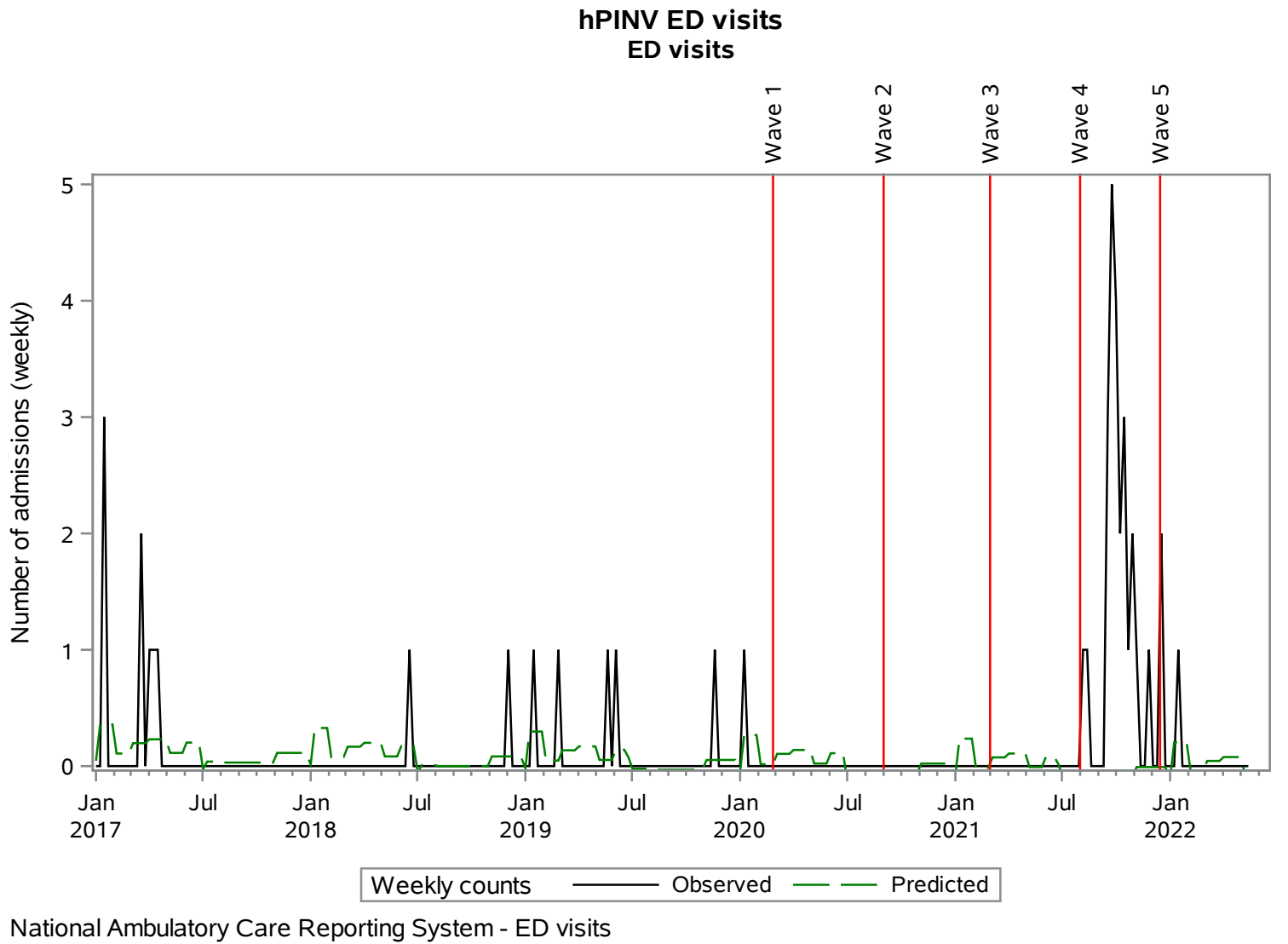

Supplement: S2 Fig — Number of hospital admissions (A) and emergency department (ED) visits associated with human parainfluenza virus (hPINV). (PDF) [file pone.0287395.s002.pdf]
