# Supplementary material for: Changes in hospitalizations and emergency department respiratory viral diagnosis trends before and during the COVID-19 pandemic in Ontario, Canada
Source: PLoS One. 2023 Jun 16;18(6):e0287395. doi: 10.1371/journal.pone.0287395 (PMC10275476; doi:10.1371/journal.pone.0287395)
Supplement: S3 Fig — Number of hospital admissions (A) and emergency department (ED) visits associated with adenovirus. (PDF) [file pone.0287395.s003.pdf]

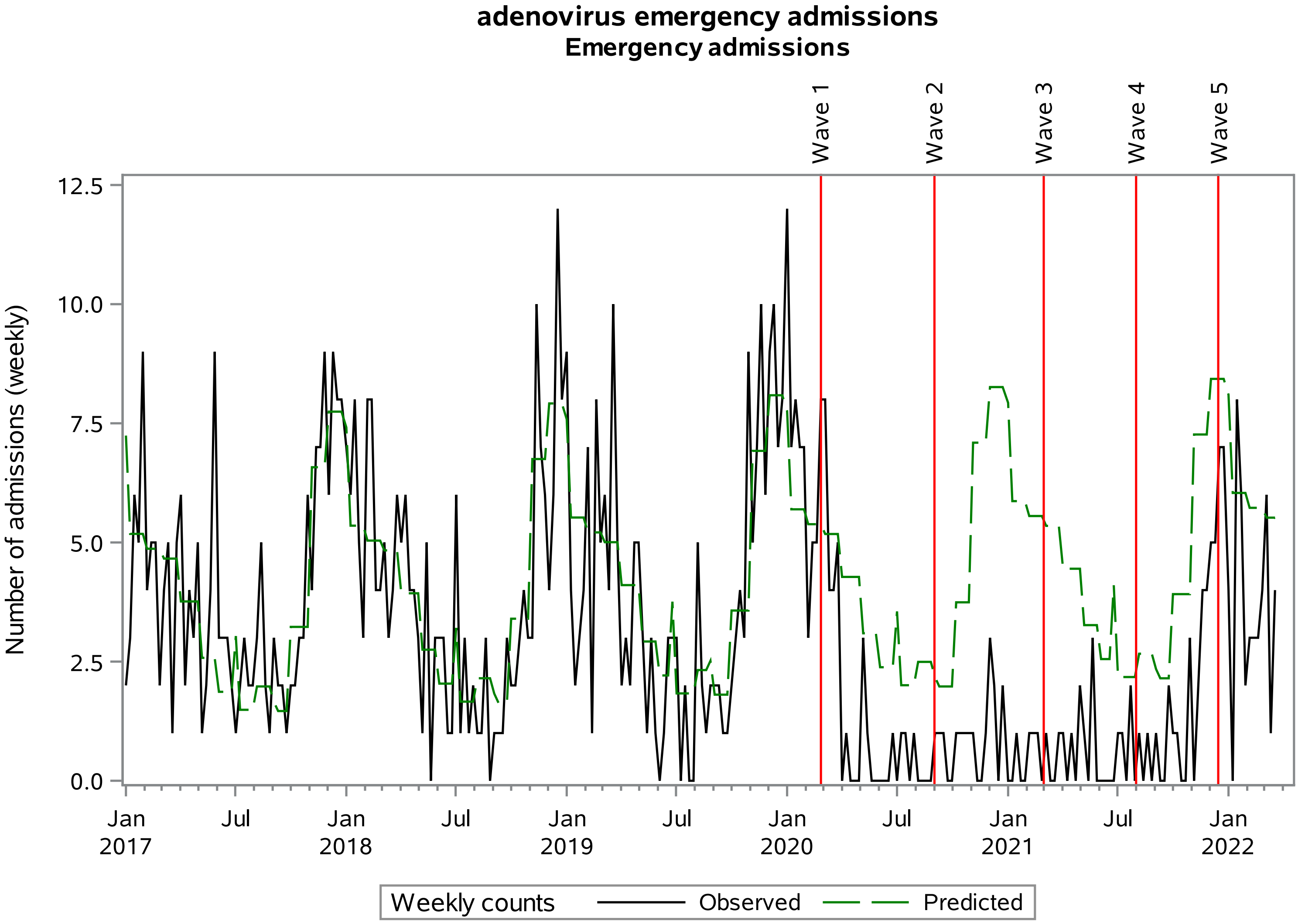

Discharge Abstract Database - emergency admissions

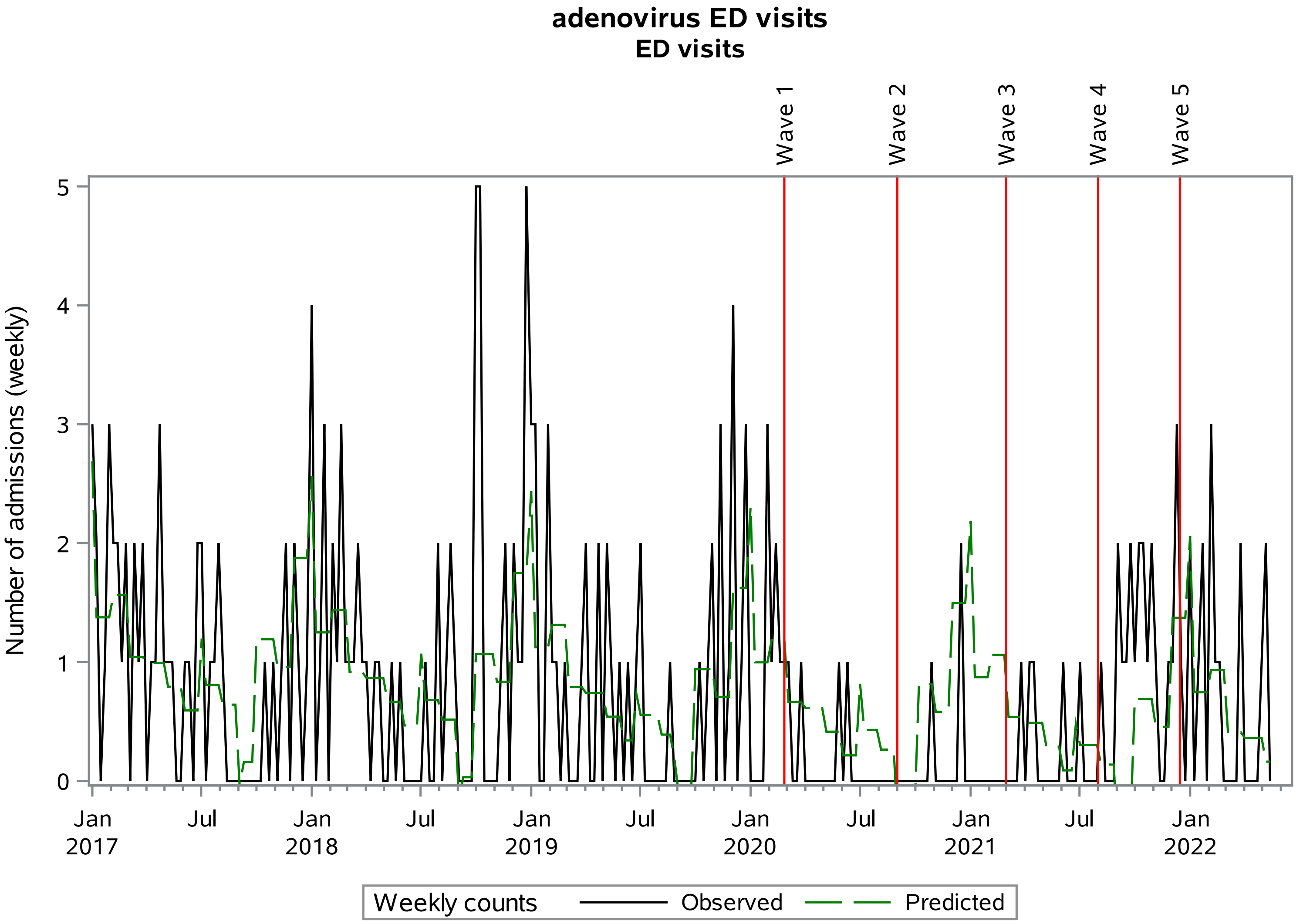

National Ambulatory Care Reporting System - ED visits
