# Supplementary material for: Changes in hospitalizations and emergency department respiratory viral diagnosis trends before and during the COVID-19 pandemic in Ontario, Canada
Source: PLoS One. 2023 Jun 16;18(6):e0287395. doi: 10.1371/journal.pone.0287395 (PMC10275476; doi:10.1371/journal.pone.0287395)
Supplement: S1 Table — Age and sex at the time of admission or emergency department visit associated with each virus. (DOCX) [file pone.0287395.s005.docx]

# S1 Table: Patient demographics by virus and hospital visit type

| **Hospital admissions** | | | | | | | | |
| --- | --- | --- | --- | --- | --- | --- | --- | --- |
|  | **Influenza**  **(N=29,894)** | **RSV**  **(N=15,768)** | **hMPV**  **(N=3,181)** | **Rhino/enterovirus**  **(N=4,039)** | **hPINV**  **(N=667)** | **Adenovirus**  **(N=829)** | **Common cold coronavirus**  **(N=722)** |  |
| Age |  |  |  |  |  |  |  |  |
| Mean (SD) | 61.6 (29.5) | 28.5 (37.1) | 53.2 (35.4) | 16.9 (27.7) | 52.7 (35.3) | 7 (15.1) | 46.6 (34.4) |  |
| Median (IQR) | 71.3 (50, 83.7) | 1.9 (0.3, 70.6) | 67.4 (5.2, 83) | 2.8 (0.9, 13.5) | 67.3 (6, 82.3) | 1.9 (1.1, 4.3) | 57.1 (4.7, 77.7) |  |
|  |  |  |  |  |  |  |  |  |
| ≤6 months | 679 (2%) | 5004 (32%) | 180 (6%) | 699 (17%) | 30 (5%) | 60 (7%) | 66 (9%) |  |
| 6.1-24 months | 1159 (4%) | 3088 (20%) | 352 (11%) | 993 (25%) | 74 (11%) | 365 (44%) | 61 (8%) |  |
| 24.1 months-5 years | 1423 (5%) | 1552 (10%) | 255 (8%) | 805 (20%) | 52 (8%) | 221 (27%) | 59 (8%) |  |
| 5.1-64.9 years | 8710 (29%) | 1526 (10%) | 714 (22%) | 1030 (26%) | 162 (24%) | 163 (20%) | 243 (34%) |  |
| ≥65 years | 17923 (60%) | 4598 (29%) | 1680 (53%) | 512 (13%) | 349 (52%) | 20 (2%) | 293 (41%) |  |
|  |  |  |  |  |  |  |  |  |
| Sex |  |  |  |  |  |  |  |  |
| Female | 15353 (51%) | 7856 (50%) | 1729 (54%) | 1793 (44%) | 365 (55%) | 364 (44%) | 339 (47%) |  |
| Male | 14541 (49%) | 7912 (50%) | 1452 (46%) | 2246 (56%) | 302 (45%) | 465 (56%) | 383 (53%) |  |
|  |  |  |  |  |  |  |  |  |
| **Emergency department visits** | | | | | | | | |
|  | **Influenza**  **(N=85,207)** | **RSV**  **(N=3,822)** | **hMPV**  **(N=8,942)** | **Rhino/enterovirus**  **(N=3,054)** | **hPINV**  **(N=43)** | **Adenovirus**  **(N=202)** | **Common cold coronavirus**  **(N=93)** |  |
| Age |  |  |  |  |  |  |  |  |
| Mean (SD) | 36.2 (24) | 5.3 (17.5) | 42.1 (24) | 7.1 (11.8) | 27.2 (36.5) | 9.6 (15.9) | 37.6 (22.5) |  |
| Median (IQR) | 33.8 (17, 54) | 0.5 (0.2, 1.5) | 42.3 (23.6, 60.9) | 2.4 (1.4, 5.8) | 3.3 (1.8, 68.6) | 3.4 (1.6, 6.8) | 40.1 (18.2, 56) |  |
|  |  |  |  |  |  |  |  |  |
| ≤6 months | 523 (1%) | 1932 (51%) | 82 (1%) | 69 (2%) | 2 (5%) | 7 (3%) | 3 (3%) |  |
| 6.1-24 months | 3224 (4%) | 1167 (31%) | 277 (3%) | 1223 (40%) | 11 (26%) | 54 (27%) | 8 (9%) |  |
| 24.1 months-5 years | 6006 (7%) | 436 (11%) | 453 (5%) | 911 (30%) | 12 (28%) | 70 (35%) | 3 (3%) |  |
| 5.1-64.9 years | 64014 (75%) | 140 (4%) | 6405 (72%) | 833 (27%) | 7 (16%) | 67 (33%) | 74 (80%) |  |
| ≥65 years | 11440 (13%) | 147 (4%) | 1725 (19%) | 18 (1%) | 11 (26%) | 4 (2%) | 5 (5%) |  |
|  |  |  |  |  |  |  |  |  |
| Sex |  |  |  |  |  |  |  |  |
| Female | 46421 (54%) | 1669 (44%) | 5383 (60%) | 1475 (48%) | 25 (58%) | 78 (39%) | 49 (53%) |  |
| Male | 38786 (46%) | 2153 (56%) | 3559 (40%) | 1579 (52%) | 18 (42%) | 124 (61%) | 44 (47%) |  |
| RSV – respiratory syncytial virus; hMPV – human metapneumovirus; hPINV – human parainfluenza virus | | | | | | | | |
