# Supplementary material for: Changes in hospitalizations and emergency department respiratory viral diagnosis trends before and during the COVID-19 pandemic in Ontario, Canada
Source: PLoS One. 2023 Jun 16;18(6):e0287395. doi: 10.1371/journal.pone.0287395 (PMC10275476; doi:10.1371/journal.pone.0287395)
Supplement: S2 Table — Factors associated with emergency department visit associated with respiratory syncytial virus during the COVID-19 era compared with the pre-COVID-19 era. (DOCX) [file pone.0287395.s006.docx]

# S2 Table: Characteristics of emergency department visits with respiratory syncytial virus

|  | Pre-COVID era (01/Jul/2017-30/Jun/2019)  (N=7530) | | COVID era (01/Jul/2021-31/Mar/2022)  (N=2262) | COVID-19 era vs. pre-COVID-19 era  (N=9592 complete case) | |
| --- | --- | --- | --- | --- | --- |
|  | **N (%)** | | **N (%)** | **OR (95% CI)^a^** | **p-value** |
| Age | |  |  |  |  |
| ≤6 months | | 1113 (51%) | 449 (45%) | 1.0 (ref) | <.0001 |
| 6.1-24 months | | 713 (32%) | 272 (27%) | 0.89 (0.73-1.08) |  |
| 24.1 months-5 years | | 214 (10%) | 197 (20%) | 1.95 (1.51-2.52) |  |
| 5.1-64.9 years | | 69 (3%) | 50 (5%) | 1.57 (1.01-2.44) |  |
| ≥65 years | | 90 (4%) | 31 (3%) | 0.92 (0.55-1.55) |  |
|  | |  |  |  |  |
| Sex | |  |  |  |  |
| Female | | 938 (43%) | 439 (44%) | 1.0 (ref) | 0.94 |
| Male | | 1261 (57%) | 560 (56%) | 1.01 (0.85-1.20) |  |
|  | |  |  |  |  |
| Month | |  |  |  |  |
| July | | 11 (1%) | <6 | 2.26 (0.69-7.41) |  |
| August | | 7 (<1%) | <6 | 6.03 (1.76-20.7) |  |
| September | | 18 (1%) | 18 (2%) | 6.49 (3.15-13.4) |  |
| October | | 45 (2%) | 82 (8%) | 9.99 (6.47-15.5) |  |
| November | | 168 (8%) | 225 (23%) | 7.23 (5.37-9.73) |  |
| December | | 614 (28%) | 479 (48%) | 4.64 (3.64-5.92) |  |
| January | | 639 (29%) | 107 (11%) | 1.0 (ref) | <.0001 |
| February | | 413 (19%) | 26 (3%) | 0.37 (0.23-0.57) |  |
| March | | 198 (9%) | 21 (2%) | 0.55 (0.33-0.92) |  |
| April | | 54 (2%) | 23 (2%) | 1.95 (1.10-3.48) |  |
| May | | 26 (1%) | 9 (1%) | 2.29 (1.02-5.14) |  |
| June | | 7 (<1%) | n/a | n/a |  |
|  | |  |  |  |  |
| Rurality | |  |  |  |  |
| Urban | | 1827 (83%) | 810 (82%) | 1.0 (ref) | 0.71 |
| Rural | | 363 (17%) | 182 (18%) | 1.05 (0.81-1.36) |  |
|  | |  |  |  |  |
| Material deprivation | |  |  |  |  |
| Lowest | | 499 (23%) | 158 (16%) | 1.0 (ref) | 0.002 |
| Mid-low | | 409 (19%) | 221 (23%) | 1.52 (1.16-2.00) |  |
| Middle | | 390 (18%) | 209 (22%) | 1.58 (1.19-2.09) |  |
| Mid-high | | 391 (18%) | 210 (22%) | 1.62 (1.20-2.17) |  |
| Highest | | 455 (21%) | 170 (18%) | 1.23 (0.89-1.70) |  |
|  | |  |  |  |  |
| Residential instability | |  |  |  |  |
| Lowest | | 392 (18%) | 187 (19%) | 1.0 (ref) | 0.42 |
| Mid-low | | 485 (23%) | 192 (20%) | 0.83 (0.63-1.09) |  |
| Middle | | 438 (20%) | 226 (23%) | 1.06 (0.80-1.41) |  |
| Mid-high | | 471 (22%) | 214 (22%) | 0.92 (0.68-1.24) |  |
| Highest | | 358 (17%) | 149 (15%) | 0.97 (0.69-1.36) |  |
|  | |  |  |  |  |
| Dependency | |  |  |  |  |
| Lowest | | 561 (26%) | 239 (25%) | 1.0 (ref) | 0.07 |
| Mid-low | | 403 (19%) | 175 (18%) | 1.18 (0.90-1.55) |  |
| Middle | | 374 (17%) | 207 (21%) | 1.47 (1.10-1.95) |  |
| Mid-high | | 390 (18%) | 160 (17%) | 1.05 (0.77-1.43) |  |
| Highest | | 416 (19%) | 187 (19%) | 1.21 (0.88-1.66) |  |
|  | |  |  |  |  |
| Ethnic diversity | |  |  |  |  |
| Lowest | | 495 (23%) | 229 (24%) | 1.0 (ref) | 0.06 |
| Mid-low | | 442 (21%) | 232 (24%) | 1.10 (0.84-1.43) |  |
| Middle | | 480 (22%) | 179 (18%) | 0.77 (0.58-1.04) |  |
| Mid-high | | 395 (18%) | 152 (16%) | 0.77 (0.56-1.07) |  |
| Highest | | 332 (15%) | 176 (18%) | 0.95 (0.67-1.34) |  |
| ^a^ Odds ratio (OR) with 95% confidence interval (CI) comparing the respiratory syncytial virus resurgence (2021/2022 season) with the pre-COVID seasons (2017/18 and 2018/19 seasons). OR are adjusted for calendar month, age at admission or emergency department visit, sex, rurality, deprivation quintile, instability quintile, dependency quintile, and ethnic diversity quintile | | | | | |
