# Supplementary material for: Changes in hospitalizations and emergency department respiratory viral diagnosis trends before and during the COVID-19 pandemic in Ontario, Canada
Source: PLoS One. 2023 Jun 16;18(6):e0287395. doi: 10.1371/journal.pone.0287395 (PMC10275476; doi:10.1371/journal.pone.0287395)
Supplement: S3 Table — Factors associated with hospitalizations associated with rhinovirus/enterovirus during the COVID-19 era compared with the pre-COVID-19 era. (DOCX) [file pone.0287395.s007.docx]

# S3 Table: Characteristics of admissions with rhinovirus/enterovirus

|  | Pre-COVID era (01/Jul/2017-30/Jun/2019)  (N=2013) | COVID era (01/Jul/2021-31/Mar/2022)  (N=521) | COVID-19 era vs. pre-COVID-19 era  (N=2465 complete case) | |
| --- | --- | --- | --- | --- |
|  | **N (%)** | **N (%)** | **OR (95% CI)^a^** | **p-value** |
| Age |  |  |  |  |
| ≤6 months | 364 (18%) | 86 (17%) | 1.0 (ref) | <.0001 |
| 6.1-24 months | 490 (24%) | 161 (31%) | 1.56 (1.13-2.14) |  |
| 24.1 months-5 years | 391 (19%) | 145 (28%) | 1.71 (1.23-2.38) |  |
| 5.1-64.9 years | 509 (25%) | 103 (20%) | 0.91 (0.65-1.28) |  |
| ≥65 years | 259 (13%) | 26 (5%) | 0.51 (0.31-0.85) |  |
|  |  |  |  |  |
| Sex |  |  |  |  |
| Female | 888 (44%) | 219 (42%) | 1.0 (ref) | 0.47 |
| Male | 1125 (56%) | 302 (58%) | 1.08 (0.87-1.34) |  |
|  |  |  |  |  |
| Month |  |  |  |  |
| July | 119 (6%) | 14 (3%) | 0.48 (0.23-1.04) |  |
| August | 108 (5%) | 40 (8%) | 1.85 (0.99-3.48) |  |
| September | 247 (125) | 76 (15%) | 1.57 (0.89-2.77) |  |
| October | 228 (115) | 97 (19%) | 2.12 (1.21-3.71) |  |
| November | 244 (12%) | 117 (22%) | 2.49 (1.43-4.33) |  |
| December | 174 (9%) | 79 (15%) | 2.25 (1.27-4.00) |  |
| January | 104 (5%) | 20 (4%) | 1.0 (ref) | <.0001 |
| February | 102 (5%) | 30 (6%) | 1.34 (0.69-2.60) |  |
| March | 138 (7%) | 48 (9%) | 1.71 (0.94-3.14) |  |
| April | 215 (11%) | n/a | n/a |  |
| May | 178 (9%) | n/a | n/a |  |
| June | 156 (8%) | n/a | n/a |  |
|  |  |  |  |  |
| Rurality |  |  |  |  |
| Urban | 1872 (94%) | 473 (91%) | 1.0 (ref) | 0.80 |
| Rural | 126 (6%) | 44 (9%) | 0.95 (0.61-1.46) |  |
|  |  |  |  |  |
| Material deprivation |  |  |  |  |
| Lowest | 433 (22%) | 123 (24%) | 1.0 (ref) | 0.03 |
| Mid-low | 342 (17%) | 75 (15%) | 0.74 (0.52-1.05) |  |
| Middle | 316 (16%) | 87 (17%) | 1.03 (0.73-1.47) |  |
| Mid-high | 388 (20%) | 97 (19%) | 1.11 (0.78-1.58) |  |
| Highest | 478 (24%) | 126 (25%) | 1.42 (0.98-2.08) |  |
|  |  |  |  |  |
| Residential instability |  |  |  |  |
| Lowest | 388 (20%) | 98 (19%) | 1.0 (ref) | 0.41 |
| Mid-low | 352 (18%) | 105 (21%) | 1.11 (0.79-1.58) |  |
| Middle | 364 (19%) | 101 (20%) | 1.04 (0.72-1.49) |  |
| Mid-high | 425 (22%) | 108 (21%) | 0.80 (0.54-1.17) |  |
| Highest | 428 (22%) | 96 (19%) | 0.87 (0.58-1.32) |  |
|  |  |  |  |  |
| Dependency |  |  |  |  |
| Lowest | 524 (27%) | 164 (32%) | 1.0 (ref) | 0.01 |
| Mid-low | 439 (22%) | 106 (21%) | 0.64 (0.47-0.88) |  |
| Middle | 323 (17%) | 86 (17%) | 0.68 (0.48-0.96) |  |
| Mid-high | 342 (17%) | 76 (15%) | 0.60 (0.42-0.86) |  |
| Highest | 329 (17%) | 76 (15%) | 0.55 (0.37-0.81) |  |
|  |  |  |  |  |
| Ethnic diversity |  |  |  |  |
| Lowest | 220 (11%) | 73 (14%) | 1.0 (ref) | 0.002 |
| Mid-low | 357 (18%) | 99 (19%) | 0.80 (0.54-1.16) |  |
| Middle | 393 (20%) | 111 (22%) | 0.74 (0.50-1.16) |  |
| Mid-high | 446 (23%) | 122 (24%) | 0.66 (0.44-1.00) |  |
| Highest | 541 (28%) | 103 (20%) | 0.43 (0.28-0.67) |  |
| ^a^ Odds ratio (OR) with 95% confidence interval (CI) comparing the respiratory syncytial virus resurgence (2021/2022 season) with the pre-COVID seasons (2017/18 and 2018/19 seasons). OR are adjusted for calendar month, age at admission or emergency department visit, sex, rurality, deprivation quintile, instability quintile, dependency quintile, and ethnic diversity quintile | | | | |
