# Supplementary material for: Changes in hospitalizations and emergency department respiratory viral diagnosis trends before and during the COVID-19 pandemic in Ontario, Canada
Source: PLoS One. 2023 Jun 16;18(6):e0287395. doi: 10.1371/journal.pone.0287395 (PMC10275476; doi:10.1371/journal.pone.0287395)
Supplement: S4 Table — Number and percent of admission episodes by ICD-10 code, by virus type. (DOCX) [file pone.0287395.s008.docx]

# S4 Table: Main diagnostic code (most responsible diagnosis) associated with admission episode, by viral infection

| **Rank** | **ICD-10 code** | **ICD-10 code description** | **N** | **%** |  |
| --- | --- | --- | --- | --- | --- |
| **Influenza virus** | | | | |  |
| **1** | J101 | Influenza with other respiratory manifestations, seasonal influenza virus identified | 7137 | 23.87 |  |
| **2** | J100 | Influenza with pneumonia, seasonal influenza virus identified | 4901 | 16.39 |  |
| **3** | J440 | Chronic obstructive pulmonary disease with acute lower respiratory infection | 1909 | 6.39 |  |
| **4** | J111 | Influenza with other respiratory manifestations, virus not identified | 1632 | 5.46 |  |
| **5** | J09 | Influenza due to identified zoonotic or pandemic influenza virus | 1360 | 4.55 |  |
| **6** | J441 | Chronic obstructive pulmonary disease with acute exacerbation, unspecified | 1343 | 4.49 |  |
| **7** | J108 | Influenza with other manifestations, seasonal influenza virus identified | 1057 | 3.54 |  |
| **8** | J110 | Influenza with pneumonia, virus not identified | 662 | 2.21 |  |
| **9** | I500 | Congestive heart failure | 618 | 2.07 |  |
| **10** | J189 | Pneumonia, unspecified | 306 | 1.02 |  |
| **11** | A419 | Sepsis, unspecified | 305 | 1.02 |  |
| **12** | J159 | Bacterial pneumonia, unspecified | 246 | 0.82 |  |
| **13** | I214 | Acute subendocardial myocardial infarction | 239 | 0.80 |  |
| **14** | J4590 | Asthma, unspecified, without stated status asthmaticus | 227 | 0.76 |  |
| **15** | J9600 | Acute respiratory failure, type 1 [hypoxic] | 207 | 0.69 |  |
| **16** | N390 | Urinary tract infection, site not specified | 202 | 0.68 |  |
| **17** | D700 | Neutropenia | 184 | 0.62 |  |
| **18** | N179 | Acute renal failure, unspecified | 177 | 0.59 |  |
| **19** | J118 | Influenza with other manifestations, virus not identified | 162 | 0.54 |  |
| **20** | J690 | Pneumonitis due to food and vomit | 148 | 0.50 |  |
| **Respiratory syncytial virus (RSV)** | | | | | |
| **1** | J210 | Acute bronchiolitis due to respiratory syncytial virus | 6776 | 42.97 |  |
| **2** | J121 | Respiratory syncytial virus pneumonia | 2708 | 17.17 |  |
| **3** | J440 | Chronic obstructive pulmonary disease with acute lower respiratory infection | 819 | 5.19 |  |
| **4** | J069 | Acute upper respiratory infection, unspecified | 583 | 3.70 |  |
| **5** | J441 | Chronic obstructive pulmonary disease with acute exacerbation, unspecified | 489 | 3.10 |  |
| **6** | J4500 | Predominantly allergic asthma without stated status asthmaticus | 307 | 1.95 |  |
| **7** | J159 | Bacterial pneumonia, unspecified | 300 | 1.90 |  |
| **8** | J205 | Acute bronchitis due to respiratory syncytial virus | 282 | 1.79 |  |
| **9** | I500 | Congestive heart failure | 236 | 1.50 |  |
| **10** | J189 | Pneumonia, unspecified | 214 | 1.36 |  |
| **11** | J22 | Unspecified acute lower respiratory infection | 168 | 1.07 |  |
| **12** | J4590 | Asthma, unspecified, without stated status asthmaticus | 146 | 0.93 |  |
| **13** | J988 | Other specified respiratory disorders | 96 | 0.61 |  |
| **14** | J101 | Influenza with other respiratory manifestations, seasonal influenza virus identified | 82 | 0.52 |  |
| **15** | J219 | Acute bronchiolitis, unspecified | 82 | 0.52 |  |
| **16** | J9600 | Acute respiratory failure, type 1 [hypoxic] | 77 | 0.49 |  |
| **17** | J690 | Pneumonitis due to food and vomit | 67 | 0.42 |  |
| **18** | N390 | Urinary tract infection, site not specified | 64 | 0.41 |  |
| **19** | D700 | Neutropenia | 58 | 0.37 |  |
| **20** | J9601 | Acute respiratory failure, type II [hypercapnic] | 54 | 0.34 |  |
| **Human metapneumovirus (hMPV)** | | | | | |
| **1** | J123 | Human metapneumovirus pneumonia | 540 | 16.98 |  |
| **2** | J2088 | Acute bronchitis due to other specified organisms | 465 | 14.62 |  |
| **3** | J440 | Chronic obstructive pulmonary disease with acute lower respiratory infection | 315 | 9.90 |  |
| **4** | J211 | Acute bronchiolitis due to human metapneumovirus | 211 | 6.63 |  |
| **5** | J069 | Acute upper respiratory infection, unspecified | 154 | 4.84 |  |
| **6** | I500 | Congestive heart failure | 131 | 4.12 |  |
| **7** | J441 | Chronic obstructive pulmonary disease with acute exacerbation, unspecified | 101 | 3.18 |  |
| **8** | J159 | Bacterial pneumonia, unspecified | 64 | 2.01 |  |
| **9** | J189 | Pneumonia, unspecified | 61 | 1.92 |  |
| **10** | J4590 | Asthma, unspecified, without stated status asthmaticus | 37 | 1.16 |  |
| **11** | U071 | Coronavirus disease 2019 [COVID-19], virus identified | 37 | 1.16 |  |
| **12** | J22 | Unspecified acute lower respiratory infection | 34 | 1.07 |  |
| **13** | D700 | Neutropenia | 31 | 0.97 |  |
| **14** | J9600 | Acute respiratory failure, type 1 [hypoxic] | 26 | 0.82 |  |
| **15** | A858 | Other specified viral encephalitis | 24 | 0.75 |  |
| **16** | J2080 | Acute bronchitis due to human metapneumovirus | 23 | 0.72 |  |
| **17** | J4500 | Predominantly allergic asthma without stated status asthmaticus | 23 | 0.72 |  |
| **18** | J690 | Pneumonitis due to food and vomit | 23 | 0.72 |  |
| **19** | N179 | Acute renal failure, unspecified | 22 | 0.69 |  |
| **20** | N390 | Urinary tract infection, site not specified | 22 | 0.69 |  |
| **Rhinovirus/enterovirus** | | | | |  |
| **1** | J069 | Acute upper respiratory infection, unspecified | 454 | 11.24 |  |
| **2** | J4500 | Predominantly allergic asthma without stated status asthmaticus | 328 | 8.12 |  |
| **3** | J218 | Acute bronchiolitis due to other specified organisms | 253 | 6.26 |  |
| **4** | B341 | Enterovirus infection, unspecified site | 227 | 5.62 |  |
| **5** | J128 | Other viral pneumonia | 215 | 5.32 |  |
| **6** | J4501 | Predominantly allergic asthma with stated status asthmaticus | 110 | 2.72 |  |
| **7** | D700 | Neutropenia | 102 | 2.53 |  |
| **8** | J440 | Chronic obstructive pulmonary disease with acute lower respiratory infection | 95 | 2.35 |  |
| **9** | J441 | Chronic obstructive pulmonary disease with acute exacerbation, unspecified | 87 | 2.15 |  |
| **10** | J210 | Acute bronchiolitis due to respiratory syncytial virus | 84 | 2.08 |  |
| **11** | J159 | Bacterial pneumonia, unspecified | 71 | 1.76 |  |
| **12** | J206 | Acute bronchitis due to rhinovirus | 69 | 1.71 |  |
| **13** | J22 | Unspecified acute lower respiratory infection | 57 | 1.41 |  |
| **14** | J219 | Acute bronchiolitis, unspecified | 56 | 1.39 |  |
| **15** | J189 | Pneumonia, unspecified | 50 | 1.24 |  |
| **16** | R509 | Fever, unspecified | 50 | 1.24 |  |
| **17** | J690 | Pneumonitis due to food and vomit | 49 | 1.21 |  |
| **18** | N390 | Urinary tract infection, site not specified | 49 | 1.21 |  |
| **19** | B348 | Other viral infections of unspecified site | 45 | 1.11 |  |
| **20** | J050 | Acute obstructive laryngitis [croup] | 40 | 0.99 |  |
| **Human parainfluenzavirus (hPINV)** | | | | | |
| **1** | J122 | Parainfluenza virus pneumonia | 307 | 46.03 |  |
| **2** | J440 | Chronic obstructive pulmonary disease with acute lower respiratory infection | 95 | 14.24 |  |
| **3** | J204 | Acute bronchitis due to parainfluenza virus | 38 | 5.70 |  |
| **4** | I500 | Congestive heart failure | 18 | 2.70 |  |
| **5** | A419 | Sepsis, unspecified | 9 | 1.35 |  |
| **6** | J159 | Bacterial pneumonia, unspecified | 9 | 1.35 |  |
| **7** | J4500 | Predominantly allergic asthma without stated status asthmaticus | 9 | 1.35 |  |
| **8** | J9600 | Acute respiratory failure, type 1 [hypoxic] | 8 | 1.20 |  |
| **9** | J100 | Influenza with pneumonia, seasonal influenza virus identified | 7 | 1.05 |  |
| **10** | J9601 | Acute respiratory failure, type II [hypercapnic] | 7 | 1.05 |  |
| **11** | J9609 | Acute respiratory failure, type unspecified | 7 | 1.05 |  |
| **12** | J9690 | Respiratory failure, unspecified, type I [hypoxic] | 6 | 0.90 |  |
| **Adenovirus** | | | | |  |
| **1** | J069 | Acute upper respiratory infection, unspecified | 136 | 16.41 |  |
| **2** | B340 | Adenovirus infection, unspecified site | 108 | 13.03 |  |
| **3** | J218 | Acute bronchiolitis due to other specified organisms | 33 | 3.98 |  |
| **4** | J210 | Acute bronchiolitis due to respiratory syncytial virus | 26 | 3.14 |  |
| **5** | R509 | Fever, unspecified | 26 | 3.14 |  |
| **6** | B349 | Viral infection, unspecified | 23 | 2.77 |  |
| **7** | J4500 | Predominantly allergic asthma without stated status asthmaticus | 18 | 2.17 |  |
| **8** | J22 | Unspecified acute lower respiratory infection | 17 | 2.05 |  |
| **9** | E860 | Dehydration | 16 | 1.93 |  |
| **10** | J189 | Pneumonia, unspecified | 16 | 1.93 |  |
| **11** | A083 | Other viral enteritis | 15 | 1.81 |  |
| **12** | H669 | Otitis media, unspecified | 14 | 1.69 |  |
| **13** | J101 | Influenza with other respiratory manifestations, seasonal influenza virus identified | 13 | 1.57 |  |
| **14** | M303 | Mucocutaneous lymph node syndrome [Kawasaki] | 13 | 1.57 |  |
| **15** | R5609 | Febrile convulsions, unspecified | 11 | 1.33 |  |
| **16** | R5601 | Complex febrile convulsions | 10 | 1.21 |  |
| **17** | A099 | Gastroenteritis and colitis of unspecified origin | 9 | 1.09 |  |
| **18** | D700 | Neutropenia | 9 | 1.09 |  |
| **19** | J159 | Bacterial pneumonia, unspecified | 9 | 1.09 |  |
| **20** | B348 | Other viral infections of unspecified site | 8 | 0.97 |  |
| **Common cold coronavirus** | | | | |  |
| **1** | U071 | Coronavirus disease 2019 [COVID-19], virus identified | 156 | 21.61 |  |
| **2** | J069 | Acute upper respiratory infection, unspecified | 58 | 8.03 |  |
| **3** | J128 | Other viral pneumonia | 37 | 5.12 |  |
| **4** | J440 | Chronic obstructive pulmonary disease with acute lower respiratory infection | 28 | 3.88 |  |
| **5** | B342 | Coronavirus infection, unspecified site | 20 | 2.77 |  |
| **6** | D700 | Neutropenia | 18 | 2.49 |  |
| **7** | J189 | Pneumonia, unspecified | 18 | 2.49 |  |
| **8** | J441 | Chronic obstructive pulmonary disease with acute exacerbation, unspecified | 18 | 2.49 |  |
| **9** | J690 | Pneumonitis due to food and vomit | 15 | 2.08 |  |
| **10** | I500 | Congestive heart failure | 14 | 1.94 |  |
| **11** | J218 | Acute bronchiolitis due to other specified organisms | 14 | 1.94 |  |
| **12** | J101 | Influenza with other respiratory manifestations, seasonal influenza virus identified | 10 | 1.39 |  |
| **13** | J210 | Acute bronchiolitis due to respiratory syncytial virus | 10 | 1.39 |  |
| **14** | J22 | Unspecified acute lower respiratory infection | 10 | 1.39 |  |
| **15** | J9600 | Acute respiratory failure, type 1 [hypoxic] | 10 | 1.39 |  |
| **16** | G419 | Status epilepticus, unspecified | 6 | 0.83 |  |
| **17** | N179 | Acute renal failure, unspecified | 6 | 0.83 |  |
