# Supplementary material for: Changes in hospitalizations and emergency department respiratory viral diagnosis trends before and during the COVID-19 pandemic in Ontario, Canada
Source: PLoS One. 2023 Jun 16;18(6):e0287395. doi: 10.1371/journal.pone.0287395 (PMC10275476; doi:10.1371/journal.pone.0287395)
Supplement: Attachment — Submitted filename: Response to reviewers - PONE-D-22-35031.docx [file pone.0287395.s009.docx]

**March 20, 2023**

**Manuscript** PONE-D-22-35031

**Title**: “Hospitalizations and emergency department visits for respiratory viruses in the time of the COVID-19 pandemic in Ontario, Canada”

**Running head:** Respiratory viruses during the COVID-19 pandemic

Dear Dr. Wu,

We thank you, the editorial team, and the reviewers for taking the time to review this manuscript. We provide point-by-point responses to the comments provided below and have tracked changes in the manuscript. All other edits were grammatical in nature to reduce the word count

Thank you for your consideration.

Sincerely,

Steven Habbous, PhD

Epidemiologist

Ontario Health (Strategic Analytics)

Email: [steven.habbous@ontariohealth.ca](mailto:steven.habbous@ontariohealth.ca)

Hospitalizations and emergency department visits for respiratory viruses in the time of the COVID-19 pandemic in Ontario, Canada

PLOS ONE

Dear Dr. Habbous,

Thank you for submitting your manuscript to PLOS ONE. We apologize for the long delay and appreciate your patience as it took some time to ascertain reviews. After careful consideration, we feel that it has merit but does not fully meet PLOS ONE’s publication criteria as it currently stands. Therefore, we invite you to submit a revised version of the manuscript that addresses the points raised during the review process.

Please submit your revised manuscript by Apr 16 2023 11:59PM. If you will need more time than this to complete your revisions, please reply to this message or contact the journal office at [plosone@plos.org](mailto:plosone@plos.org). When you're ready to submit your revision, log on to <https://www.editorialmanager.com/pone/> and select the 'Submissions Needing Revision' folder to locate your manuscript file.

Please include the following items when submitting your revised manuscript:

- A rebuttal letter that responds to each point raised by the academic editor and reviewer(s). You should upload this letter as a separate file labeled 'Response to Reviewers'.
- A marked-up copy of your manuscript that highlights changes made to the original version. You should upload this as a separate file labeled 'Revised Manuscript with Track Changes'.
- An unmarked version of your revised paper without tracked changes. You should upload this as a separate file labeled 'Manuscript'.

If you would like to make changes to your financial disclosure, please include your updated statement in your cover letter. Guidelines for resubmitting your figure files are available below the reviewer comments at the end of this letter.

If applicable, we recommend that you deposit your laboratory protocols in protocols.io to enhance the reproducibility of your results. Protocols.io assigns your protocol its own identifier (DOI) so that it can be cited independently in the future. For instructions see: [https://journals.plos.org/plosone/s/submission-guidelines#loc-laboratory-protocols](https://can01.safelinks.protection.outlook.com/?url=https%3A%2F%2Fjournals.plos.org%2Fplosone%2Fs%2Fsubmission-guidelines%23loc-laboratory-protocols&data=05%7C01%7Csteven.habbous%40ontariohealth.ca%7C769ab4c103df4032215908db1b2e1907%7C4ef96c5cd83f466ba478816a5bb4af62%7C0%7C0%7C638133657179886982%7CUnknown%7CTWFpbGZsb3d8eyJWIjoiMC4wLjAwMDAiLCJQIjoiV2luMzIiLCJBTiI6Ik1haWwiLCJXVCI6Mn0%3D%7C3000%7C%7C%7C&sdata=FcAu2wPIFWDvewQr6eDjocPBsQQ8rBb%2FD6rEnY5Yk5E%3D&reserved=0). Additionally, PLOS ONE offers an option for publishing peer-reviewed Lab Protocol articles, which describe protocols hosted on protocols.io. Read more information on sharing protocols at [https://plos.org/protocols?utm_medium=editorial-email&utm_source=authorletters&utm_campaign=protocols](https://can01.safelinks.protection.outlook.com/?url=https%3A%2F%2Fplos.org%2Fprotocols%3Futm_medium%3Deditorial-email%26utm_source%3Dauthorletters%26utm_campaign%3Dprotocols&data=05%7C01%7Csteven.habbous%40ontariohealth.ca%7C769ab4c103df4032215908db1b2e1907%7C4ef96c5cd83f466ba478816a5bb4af62%7C0%7C0%7C638133657179886982%7CUnknown%7CTWFpbGZsb3d8eyJWIjoiMC4wLjAwMDAiLCJQIjoiV2luMzIiLCJBTiI6Ik1haWwiLCJXVCI6Mn0%3D%7C3000%7C%7C%7C&sdata=pqBtpTikkOMJHkeO9KQtUAMlZEY0Ehptpj%2BFD8YQL3g%3D&reserved=0).

We look forward to receiving your revised manuscript.

Kind regards,

Andrew G Wu
Academic Editor
PLOS ONE

Journal Requirements:

When submitting your revision, we need you to address these additional requirements.

1. Please ensure that your manuscript meets PLOS ONE's style requirements, including those for file naming. The PLOS ONE style templates can be found at 
<https://journals.plos.org/plosone/s/file?id=wjVg/PLOSOne_formatting_sample_main_body.pdf> and 
<https://journals.plos.org/plosone/s/file?id=ba62/PLOSOne_formatting_sample_title_authors_affiliations.pdf>

**Response:** We have reviewed the requirements and made appropriate edits.

2. In your Data Availability statement, you have not specified where the minimal data set underlying the results described in your manuscript can be found. PLOS defines a study's minimal data set as the underlying data used to reach the conclusions drawn in the manuscript and any additional data required to replicate the reported study findings in their entirety. All PLOS journals require that the minimal data set be made fully available. For more information about our data policy, please see <http://journals.plos.org/plosone/s/data-availability>.

Upon re-submitting your revised manuscript, please upload your study’s minimal underlying data set as either Supporting Information files or to a stable, public repository and include the relevant URLs, DOIs, or accession numbers within your revised cover letter. For a list of acceptable repositories, please see <http://journals.plos.org/plosone/s/data-availability#loc-recommended-repositories>. Any potentially identifying patient information must be fully anonymized.

Important: If there are ethical or legal restrictions to sharing your data publicly, please explain these restrictions in detail. Please see our guidelines for more information on what we consider unacceptable restrictions to publicly sharing data: <http://journals.plos.org/plosone/s/data-availability#loc-unacceptable-data-access-restrictions>. Note that it is not acceptable for the authors to be the sole named individuals responsible for ensuring data access.

We will update your Data Availability statement to reflect the information you provide in your cover letter.

**Response:** We have added the data availability statement to the cover letter. Due to legal and privacy issues, we are prohibited from making the data publicly available. A non-author point of contact is the Ontario Health Research Office ([OH-CCO_Research@ontariohealth.ca](mailto:OH-CCO_Research@ontariohealth.ca)).

3. Please note that in order to use the direct billing option the corresponding author must be affiliated with the chosen institute. Please either amend your manuscript to change the affiliation or corresponding author, or email us at [plosone@plos.org](mailto:plosone@plos.org) with a request to remove this option.

**Response:** This is my first time submitting to a PLoS journal and have no funding for this work. However, my library contacts at Western University assure me that there should be no issue given the funding agreement between PLoS and Western University.

[Note: HTML markup is below. Please do not edit.]

Reviewers' comments:

Reviewer's Responses to Questions

**Comments to the Author**

1. Is the manuscript technically sound, and do the data support the conclusions?

The manuscript must describe a technically sound piece of scientific research with data that supports the conclusions. Experiments must have been conducted rigorously, with appropriate controls, replication, and sample sizes. The conclusions must be drawn appropriately based on the data presented.

Reviewer #1: Partly

Reviewer #2: Yes

2. Has the statistical analysis been performed appropriately and rigorously?

Reviewer #1: Yes

Reviewer #2: I Don't Know

3. Have the authors made all data underlying the findings in their manuscript fully available?

The [PLOS Data policy](https://can01.safelinks.protection.outlook.com/?url=http%3A%2F%2Fwww.plosone.org%2Fstatic%2Fpolicies.action%23sharing&data=05%7C01%7Csteven.habbous%40ontariohealth.ca%7C769ab4c103df4032215908db1b2e1907%7C4ef96c5cd83f466ba478816a5bb4af62%7C0%7C0%7C638133657179886982%7CUnknown%7CTWFpbGZsb3d8eyJWIjoiMC4wLjAwMDAiLCJQIjoiV2luMzIiLCJBTiI6Ik1haWwiLCJXVCI6Mn0%3D%7C3000%7C%7C%7C&sdata=RM2q29ZezOAsLjzNl7OtFiFWk0FWqAbZmvD0sSEbz64%3D&reserved=0) requires authors to make all data underlying the findings described in their manuscript fully available without restriction, with rare exception (please refer to the Data Availability Statement in the manuscript PDF file). The data should be provided as part of the manuscript or its supporting information, or deposited to a public repository. For example, in addition to summary statistics, the data points behind means, medians and variance measures should be available. If there are restrictions on publicly sharing data—e.g. participant privacy or use of data from a third party—those must be specified.

Reviewer #1: No

Reviewer #2: Yes

4. Is the manuscript presented in an intelligible fashion and written in standard English?

PLOS ONE does not copyedit accepted manuscripts, so the language in submitted articles must be clear, correct, and unambiguous. Any typographical or grammatical errors should be corrected at revision, so please note any specific errors here.

Reviewer #1: Yes

Reviewer #2: Yes

5. Review Comments to the Author

Please use the space provided to explain your answers to the questions above. You may also include additional comments for the author, including concerns about dual publication, research ethics, or publication ethics. (Please upload your review as an attachment if it exceeds 20,000 characters)

**Reviewer 1 comments**

**Reviewer #1:**

Thank you for the opportunity to review this manuscript. Habbous et al use administrative code data for a number of common community respiratory viruses in Ontario Canada and describe the change in trends before and during the COVID-19 pandemic. They find that there are important differences by viral species that reflect similar findings reported in other locations. The topic of COVID-19 pandemic effects on common respiratory viruses has been well documented in different locations but with slight differences that are likely affected by differences in public health interventions, community attitudes and behaviors towards COVID-19 as well as climate. As such, additional publications such as this I believe still has relevance and would be of interest to the readers of PLOSOne. However, as the authors highlight, the biggest limitation with this study is the use of administrative codes. Rather than dive further into the limitations of this, I have some suggestions below. Additionally, I have provide comments, questions and suggestions that I hope will be helpful to the authors as they consider revision.

Major Consideration

**Comment #1:** As mentioned, the biggest limitation to this study is the use of administrative codes. Testing surveillance already has limitations caused by pandemic related health seeking behaviors and supply chain issues. Administrative codes would have even more bias given that it relies on the coder to accurately depict the type of respiratory viral infection. I would strongly suggest that the authors be as upfront about this limitation as possible and put it in the title. I suggest “Changes in Hospitalization and Emergency Department Respiratory Viral Diagnosis Trends Before and During the COVID-19 Pandemic, Ontario, Canada.”

**Response:** Thank you for the suggestion, we have changed the title accordingly. We agree that the wording of the new title is more aligned with the paper’s methods.

**Comment #2:** In the introduction, the authors highlight a number of reasons why there may have been changes in respiratory viral circulation during the pandemic but failed to mention the possibility of viral interference. I suggest including this and also adding a citation. I also suggest that testing behaviors due to heightened awareness of respiratory viruses or in some cases prioritizing COVID-19 may have influenced diagnoses of other respiratory viruses. Reagent limitations and supply chain issues also played a role in these.

**Response:** These are all excellent points and we have listed them as possible reasons for reduced hospital activity in the introduction. This part of the introduction now reads as follows:

**“**Since the start of the COVID-19 pandemic, the number of people affected by influenza, RSV, and the broader array of respiratory viruses decreased in many countries.^4–9^ Although this may be partially driven by viral interference caused by COVID-19 infection, reduced testing due to reagent shortages, and laboratory burden, the main drivers are likely modifications in human behavior as a result of public health measures (e.g. restrictions, lockdowns) and individual risk-reduction tactics (e.g. mask-wearing, avoiding crowded spaces, increased hand hygiene) to combat the COVID-19 pandemic.^10,11^”

We have also added to the discussion the following:

“It is also possible that viral interference (e.g. infection with COVID-19 temporarily guarded against other infections) may have prevented some hospitalizations related to influenza or RSV, although perhaps less with rhinovirus.”

**Comment #3:** A number of studies on this topic have been published that have provided a comprehensive overview of the literature out there. I would suggest also including this study in the introduction: <https://www.nature.com/articles/s41579-022-00807-9> and it can also be added to the discussion as this paper includes a discussion on hospitalization and ED visits.

**Response:** This is a very thorough review. We have added this citation to the introduction upon listing the aforementioned reasons for changes in respiratory viral circulation. We also cite this review in the discussion upon expounding on rhinovirus/enterovirus.

**Comment #4:** On page 7 when the authors describe the changes in age-groups among children, I suggest also pointing out that the proportion of adults > 65 + remained suppressed in contrast to the increase in pediatric cases during the pandemic. It would be worth then to elaborate on this in the discussion. Falsey et al attributed this change to the return of children to school and the ongoing mask use in those 65 years and older given the higher risk for severe outcomes related to COVID-19: <https://academic.oup.com/jid/article/227/1/83/6783142>

**Response:** We have added the following to the discussion, referencing this study:

**“**Admissions with RSV or rhinovirus/enterovirus were less likely to comprise of patients age 65+ and more likely to comprise of young children (ages 2-5), which may be to continued adherence to various public health measures such as masking among the elderly amidst easing restrictions (Falsey et al).”

**Comment #5:** In the discussion, the authors provide highlight that rhino/enteroviruses may transmit more efficiently by fomites and surface contamination as the reason for their increases later in the pandemic. However, other reasons exist as described in the discussion here: <https://academic.oup.com/jid/article/226/Supplement_3/S304/6617600?searchresult=1>

**Response:** The discussion from this article was very helpful. We have added the following to the discussion, adding appropriate references throughout:

**“**It is possible that viruses such as rhinoviruses and enteroviruses transmit more efficiently than other respiratory viruses, explaining their rising impacts later in the pandemic. Rhinoviruses are non-enveloped viruses, prolonging their survival on inanimate surfaces and enabling transmission by fomites and surface contact (PMC4462923, 30373527). Masking is also less effective against rhinoviruses than influenza (PMC8238571).”

**Comment #6:** It is not clear to me how the authors established the predicted levels of hospital admissions and emergency department visits in Figure 1 and 2. This should be described in the methods.

**Response:** Thank you for noticing this. We have added the following to the “Statistics and privacy” subsection of the methods:

“The predicted levels of admissions or emergency department visits were constructed using a linear regression on the weekly number of visits between January 2017 and February 2020 using calendar year, month, and holiday weeks as predictors. This was then extrapolated into the COVID-19 era.”

**Comment 7:** Other viruses were highlighted in the results other than influenza, RSV, rhino/enterovirus. Suggest putting the study results in context to what is published on others such as HPIV, HMV, Adenovirus.

**Response:** Great suggestion. We have added the following to the end of the discussion before the limitations:

“We did not observe a resurgence of hMPV, in contrast to reports from Western Australia, Israel, and the Netherlands.(PMC9612024, 35472530, PMC8847107) Our study also found a return of hPINV earlier than expected, and a return to pre-pandemic admissions with seasonal coronavirus and adenovirus-associated, although the numbers of events were small. Similar increases were observed in England.( PMC8591975)”

Other considerations
**Comment 8:** Rather than referring to endemic coronaviruses as “seasonal coronavirus” I suggest using the term common cold coronaviruses: <https://pubmed.ncbi.nlm.nih.gov/36052654/>

**Response:** We have changed the language throughout (and cited this paper).

**Comment 9:** Page 7 there is a typo when the authors compare the percentages of hospitalized children before and during the pandemic (should be 8% instead of 32%).

**Response:** Good catch, we have made this correction.

**Comment 10:** Figure 1 – subtitles should be hospital admissions and not emergency admissions.

**Response:** Thank you for the suggestion. We have removed the subtitles from the figures, as these have become redundant

**Comment 11:** Table 2 – age groups should not have overlapping ages (e.g. 0-6 months, 6-24 months)

**Response:** We have specified the cut-points for Table 2 and the supplementary tables.

**Comment 12:** eFigure S1 should indicate whether these data are before, during the pandemic or over the study period.

**Response:** These supplementary figures were over the entire study period. We have added a caption to clarify this.

**Reviewer #2 comments**

To improve the quality of the manuscript, I would suggest the authors:

**Comment 1:** to include the main reason and relative % of hospitalization

**Response:** Thank you for the suggestion. We have added a supplementary table to indicate, for each virus, the most responsible diagnosis associated with that admission (eTable S3).

**Comment2:** to consider some variables (such as intensive care unit occupation or oxygen supplementation): they may influence hospitalization length and contribute to a high hospitalization costs

**Response:** This is a great suggestion. We have added total admission length-of-stay, use of ICU, and provision of respiratory ventilation or oxygenation as outcomes, by viral type (new Table 3). We have added the following to the new “outcomes” subsection of the methods:

“During the admission episode, we examined the utilization of intensive care units (ICU) (special care unit codes 10, 30, 70, or 80) and use of respiratory support (1GZ30, 1GZ31, 1GZ32, 1GZ35, 1GZ38). Total length of stay was computed, in days, at the level of the entire admission episode.”

We have also added the following to the new “outcomes” subsection of the results:

Use of ICU during the admission episode was highest for hPINV (23%), followed by rhinovirus/enterovirus (18%), hMPV (14.6%), influenza (11.3%), adenovirus (9.7%), and RSV (9.2%) (Table 3). The high rate of ICU use also observed among common cold coronaviruses (18%) may be driven by misclassification with the novel COVID-19 (eFigure S4; eTable S3). Respiratory support during the admission episode was highest for admissions with common-cold coronavirus (17%) and hPINV (16%), followed by hMPV (10%), rhinovirus/enterovirus (8.9%), influenza virus (7.5%), and RSV (5.8%). Total length-of-stay was highest for hPINV (median 6.6 days), hMPV (median 4.8 days), and influenza virus (median 4.7 days).

ICU use in the COVID-19 era was more likely for admissions with hMPV [OR 1.89 (1.13-3.16)]. Use of respiratory support was trending higher in the COVID era for hMPV [OR 1.64 (0.91-2.94)], but decreased for RSV [OR 0.61 (0.47-0.78)] and rhinovirus/enterovirus [OR 0.53 (0.34-0.82)] (Table 3).

**Comment3:** to include in the discussion a comparison with other reports ( for example: doi: 10.3390/ijerph192315455) supporting the need of protection in infants and elderly ( doi: 10.1186/s12982-021-00104-5 doi: 10.1056/NEJMoa2116154)

**Response:** Thank you for these references, we have added these references to the discussion: “Alleviating strain on the healthcare system may include public health campaigns to promote influenza vaccination, further uptake of anti-RSV vaccination (maternal or among higher-risk infants and children)^43^, explore RSV vaccination for older adults.^44^”

6. PLOS authors have the option to publish the peer review history of their article ([what does this mean?](https://can01.safelinks.protection.outlook.com/?url=https%3A%2F%2Fjournals.plos.org%2Fplosone%2Fs%2Feditorial-and-peer-review-process%23loc-peer-review-history&data=05%7C01%7Csteven.habbous%40ontariohealth.ca%7C769ab4c103df4032215908db1b2e1907%7C4ef96c5cd83f466ba478816a5bb4af62%7C0%7C0%7C638133657179886982%7CUnknown%7CTWFpbGZsb3d8eyJWIjoiMC4wLjAwMDAiLCJQIjoiV2luMzIiLCJBTiI6Ik1haWwiLCJXVCI6Mn0%3D%7C3000%7C%7C%7C&sdata=ycnW%2FkmZbqKcRMvs9kJdb5yjpaLj%2FIi6aO7p%2FE65H18%3D&reserved=0)). If published, this will include your full peer review and any attached files.


If you choose “no”, your identity will remain anonymous but your review may still be made public.


**Do you want your identity to be public for this peer review?** For information about this choice, including consent withdrawal, please see our [Privacy Policy](https://can01.safelinks.protection.outlook.com/?url=https%3A%2F%2Fwww.plos.org%2Fprivacy-policy&data=05%7C01%7Csteven.habbous%40ontariohealth.ca%7C769ab4c103df4032215908db1b2e1907%7C4ef96c5cd83f466ba478816a5bb4af62%7C0%7C0%7C638133657179886982%7CUnknown%7CTWFpbGZsb3d8eyJWIjoiMC4wLjAwMDAiLCJQIjoiV2luMzIiLCJBTiI6Ik1haWwiLCJXVCI6Mn0%3D%7C3000%7C%7C%7C&sdata=rDNLQhCMHU%2FfnNmE%2BWAY2WsNyMrxCSJrAc8eyYaANzE%3D&reserved=0).

Reviewer #1: No

Reviewer #2: **Yes:** Elena Bozzola
