# Supplementary material for: Changes in hospitalizations and emergency department respiratory viral diagnosis trends before and during the COVID-19 pandemic in Ontario, Canada
Source: PLoS One. 2023 Jun 16;18(6):e0287395. doi: 10.1371/journal.pone.0287395 (PMC10275476; doi:10.1371/journal.pone.0287395)
Supplement: Attachment — Submitted filename: Response to reviewers - PONE-D-22-35031.R1.docx [file pone.0287395.s010.docx]

**May 31, 2023**

**Manuscript** PONE-D-22-35031

**Title**: “Hospitalizations and emergency department visits for respiratory viruses in the time of the COVID-19 pandemic in Ontario, Canada”

**Running head:** Respiratory viruses during the COVID-19 pandemic

Dear Dr. Wu,

We thank you, the editorial team, and the reviewers for taking the time to review this manuscript. We provide point-by-point responses to the comments provided below and have tracked changes in the manuscript. Any changes to the reference list included 1) updating recent publications with their volume, issue, and page number if available; and 2) removing 1 duplicate reference.

Thank you for your consideration.

Sincerely,

Steven Habbous, PhD

Epidemiologist

Ontario Health (Strategic Analytics)

Email: [steven.habbous@ontariohealth.ca](mailto:steven.habbous@ontariohealth.ca)

Hospitalizations and emergency department visits for respiratory viruses in the time of the COVID-19 pandemic in Ontario, Canada

PLOS ONE

Dear Dr. Habbous,

Thank you for submitting your manuscript to PLOS ONE. After careful consideration, we feel that it has merit but does not fully meet PLOS ONE’s publication criteria as it currently stands. Therefore, we invite you to submit a revised version of the manuscript that addresses the points raised during the review process.

I'm also pleased to report that I am happy to accept your manuscript for publication once the minor revisions have been addressed.

Please submit your revised manuscript by Jul 15 2023 11:59PM. If you will need more time than this to complete your revisions, please reply to this message or contact the journal office at [plosone@plos.org](mailto:plosone@plos.org). When you're ready to submit your revision, log on to <https://www.editorialmanager.com/pone/> and select the 'Submissions Needing Revision' folder to locate your manuscript file.

Please include the following items when submitting your revised manuscript:

- A rebuttal letter that responds to each point raised by the academic editor and reviewer(s). You should upload this letter as a separate file labeled 'Response to Reviewers'.
- A marked-up copy of your manuscript that highlights changes made to the original version. You should upload this as a separate file labeled 'Revised Manuscript with Track Changes'.
- An unmarked version of your revised paper without tracked changes. You should upload this as a separate file labeled 'Manuscript'.

If you would like to make changes to your financial disclosure, please include your updated statement in your cover letter. Guidelines for resubmitting your figure files are available below the reviewer comments at the end of this letter.

If applicable, we recommend that you deposit your laboratory protocols in protocols.io to enhance the reproducibility of your results. Protocols.io assigns your protocol its own identifier (DOI) so that it can be cited independently in the future. For instructions see: [https://journals.plos.org/plosone/s/submission-guidelines#loc-laboratory-protocols](https://can01.safelinks.protection.outlook.com/?url=https%3A%2F%2Fjournals.plos.org%2Fplosone%2Fs%2Fsubmission-guidelines%23loc-laboratory-protocols&data=05%7C01%7Csteven.habbous%40ontariohealth.ca%7C3e773503e64e42ed9cb308db61f2774d%7C4ef96c5cd83f466ba478816a5bb4af62%7C0%7C0%7C638211466240970036%7CUnknown%7CTWFpbGZsb3d8eyJWIjoiMC4wLjAwMDAiLCJQIjoiV2luMzIiLCJBTiI6Ik1haWwiLCJXVCI6Mn0%3D%7C3000%7C%7C%7C&sdata=5VyL9SJPAMt7pJRReMp19GSHCX6v3tlelEIQruTQo4E%3D&reserved=0). Additionally, PLOS ONE offers an option for publishing peer-reviewed Lab Protocol articles, which describe protocols hosted on protocols.io. Read more information on sharing protocols at [https://plos.org/protocols?utm_medium=editorial-email&utm_source=authorletters&utm_campaign=protocols](https://can01.safelinks.protection.outlook.com/?url=https%3A%2F%2Fplos.org%2Fprotocols%3Futm_medium%3Deditorial-email%26utm_source%3Dauthorletters%26utm_campaign%3Dprotocols&data=05%7C01%7Csteven.habbous%40ontariohealth.ca%7C3e773503e64e42ed9cb308db61f2774d%7C4ef96c5cd83f466ba478816a5bb4af62%7C0%7C0%7C638211466240970036%7CUnknown%7CTWFpbGZsb3d8eyJWIjoiMC4wLjAwMDAiLCJQIjoiV2luMzIiLCJBTiI6Ik1haWwiLCJXVCI6Mn0%3D%7C3000%7C%7C%7C&sdata=acGtE6Xxs4mNPpw0SYnAbu0JWgrgM%2F6gQcBrEjCAPj8%3D&reserved=0).

We look forward to receiving your revised manuscript.

Kind regards,

Andrew G Wu
Academic Editor
PLOS ONE

Journal Requirements:

Please review your reference list to ensure that it is complete and correct. If you have cited papers that have been retracted, please include the rationale for doing so in the manuscript text, or remove these references and replace them with relevant current references. Any changes to the reference list should be mentioned in the rebuttal letter that accompanies your revised manuscript. If you need to cite a retracted article, indicate the article’s retracted status in the References list and also include a citation and full reference for the retraction notice.

Please use the space provided to explain your answers to the questions above. You may also include additional comments for the author, including concerns about dual publication, research ethics, or publication ethics. (Please upload your review as an attachment if it exceeds 20,000 characters)

**Reviewer 1 comments**

Thank you for taking the time to consider my comments and suggestions. I appreciate all that was done to address my concerns. A couple of minor points:

**Comment #1:** In the introduction, detection of respiratory viruses during the pandemic may have also been affected by testing behavior. Early on, people may have feared going to the clinic for testing or later, people no longer cared to know what their diagnosis was unless they were sick enough to be hospitalized.

**Response:** Thank you for the comment, we have added this possibility to the introduction along with a relevant reference (#5; Moore et al).

**Comment #2:** All instances in which you're referring to the infection, it's probably more accurate to say SARS-CoV-2 infection. When referring to the disease you can use COVID-19. Suggest not using COVID-19 infection.

**Response:** Great point. We went through the manuscript to ensure whenever we’re referring to the infection, we are using SARS-CoV-2 instead of COVID-19. When speaking more generally (e.g. the COVID-19 era; the COVID-19 pandemic), we retained the use of COVID-19.

**Comment #3:** Table 2 - The age group overlap is still a little confusing to me. I am not familiar with the use of parentheses and brackets to represent inclusivity or exclusivity. I suspect other readers may also not be familiar with this. Please adjust to be clear or be clear in the footnotes about how these are used.

**Response:** Great point. We went through the manuscript to ensure whenever we’re referring to the infection, we are using SARS-CoV-2 instead of COVID-19. When speaking more generally (e.g. the COVID-19 era; the COVID-19 pandemic), we retained the use of COVID-19.

**Comment #4:** If you are short on word count, you could consider abbreviating common cold coronavirus as ccCoV

**Reviewer 2 comments**

Authors provided an answer to my concern. The manuscript is suitable for publication in the present form
